# The extracellular matrix in cancer-associated fibrosis: molecular mechanisms and clinical relevance

**DOI:** 10.1172/jci.insight.202316

**Published:** 2026-07-08

**Authors:** Romain Desert, Orlando Musso, Thomas F. Baumert

**Affiliations:** 1 University of Strasbourg, Inserm, Institute for Translational Medicine and Liver Disease, UMR_S1110, Strasbourg, France.; 2 Inserm, University of Rennes, INRAe, UMR 1317, Nutrition, Métabolismes et Cancer, Rennes, France.; 3 IHU Strasbourg, Strasbourg, France.; 4 Gastroenterology and Hepatology Service, Strasbourg University Hospitals, Strasbourg, France.; 5 Institut Universitaire de France, Paris, France.

## Abstract

The ECM is a dynamic component of the tumor microenvironment with a critical role in cancer progression, invasion, metastasis, immune exclusion, and response to therapy. Recent advances in proteomic analyses investigating the insoluble ECM fractions (termed “matrisome analysis”), along with single-cell RNA sequencing and spatial transcriptomics, have revealed cancer-specific patterns of ECM remodeling. These studies have identified a panel of recurrently upregulated ECM proteins, including annexin A1, fibrillin-1, fibronectin, periostin, and tenascin-C, actively contributing to tumor growth, invasion, angiogenesis, and immune exclusion. The expression of the cancer-associated ECM is largely driven by cancer-associated fibroblasts (CAFs), whose molecular diversity has been dissected through single-cell profiling and consolidated in emerging CAF atlases across cancers. By investigating the matrisome composition and CAF heterogeneity, these studies have unraveled the pivotal role of the stroma in shaping tumor biology. Based on these discoveries, ECM proteins and CAFs are now being explored as biomarkers and therapeutic targets. Future integration of multi-omics datasets with clinical outcomes will help to translate these insights into novel biomarkers for patient stratification and stroma-directed therapeutic interventions.

## Introduction

A large proportion of cancers arise in a fibroinflammatory background. For example, in oral squamous cell carcinoma, submucosal fibrosis increases cancer risk by almost 20-fold (1). In the lung, idiopathic pulmonary fibrosis increases cancer risk by 6-fold (2), which is similar to the increase in risk of breast cancer for women showing dense radiological patterns of fibrosis (3). In the liver, more than 80% of hepatocellular carcinomas (HCCs) develop in patients with variable extents of fibroinflammatory liver diseases, making severe fibrosis and its end-stage condition, cirrhosis, the main risk factor of HCC, with a relative risk of almost 100-fold compared with patients without fibrosis (4).

Carcinogenesis is supported by inflammation and desmoplasia. Desmoplasia, also known as “stromal reaction,” starts when growth factors lead to increased vascular permeability, permitting extravasation of plasma and coagulation factors, which in turn results in the local deposition of a fibrin gel that serves as a provisional ECM that favors cell migration (5). As this gel matrix is a potent chemoattractant for inflammatory cells, it is progressively replaced by granulation tissue composed of endothelial cells and myofibroblasts (6). The diverse cell types migrating and proliferating through this gel matrix secrete and deposit ECM proteins, including collagens, noncollagenous glycoproteins and proteoglycans, ultimately leading to the dense scar tissue known as desmoplasia (5).

In a seminal article (7), H. Dvorak described tumors as *“*wounds that do not heal,*”* because once the processes of inflammation and desmoplasia are set off by cancer cells, they never stop, so long as cancer cells remain.

Ongoing formation of granulation tissue and desmoplasia are key events in remodeling the tumor landscape, that provide growth factors, immune cell regulators, and structural support for cancer cell migration and invasion. Coevolution of cancer cells and their supporting fibroinflammatory stroma circumvents antitumor immunity through three independent yet interconnected mechanisms: immune evasion, immune exclusion and immune exhaustion. Immune evasion is the ability of cancer cells to escape recognition and destruction by the immune system through loss of antigen presentation, secretion of immunosuppressive factors, or activation of inhibitory checkpoints (8). Immune exclusion refers to the spatial segregation of immune cells from tumor cell clusters, often due to physical or functional barriers (e.g., fibrosis) in the tumor microenvironment (TME) (9). Immune exhaustion is a functional state of immune cells, particularly CD4^+^ and CD8^+^ T cells, characterized by progressive loss of effector functions due to chronic stimulation *(*e.g., persistence of viral, parasitic, and/or tumor antigens). Thus, despite the expression of activation markers, exhausted T cells exhibit reduced effector functions (e.g., cytokine production, cytotoxicity), incomplete differentiation, impaired trafficking to tumor sites from lymph nodes, and upregulated inhibitory receptors (e.g., PD-1; CTLA-4) as well as reprogrammed metabolism and epigenetics (10–12). These three mechanisms usually coexist within the TME. While immune exhaustion is typically the main target of immunotherapies, immune evasion and immune exclusion can be important mechanisms of therapy resistance and provide additional therapeutic opportunities (13, 14).

In this Review, we discuss recent advances in elucidating how desmoplasia shapes cancer biology, highlighting ECM components commonly and specifically overexpressed across cancers. We outline key candidate mediators in cancer-specific ECM remodeling associated with tumor onset and progression. We also discuss the heterogeneity of cancer-associated fibroblast (CAF) subpopulations in cancer and the potential of CAF-targeting strategies for biomarkers and therapy.

Composition and function of the ECM in desmoplasia.

**Figure 1:**
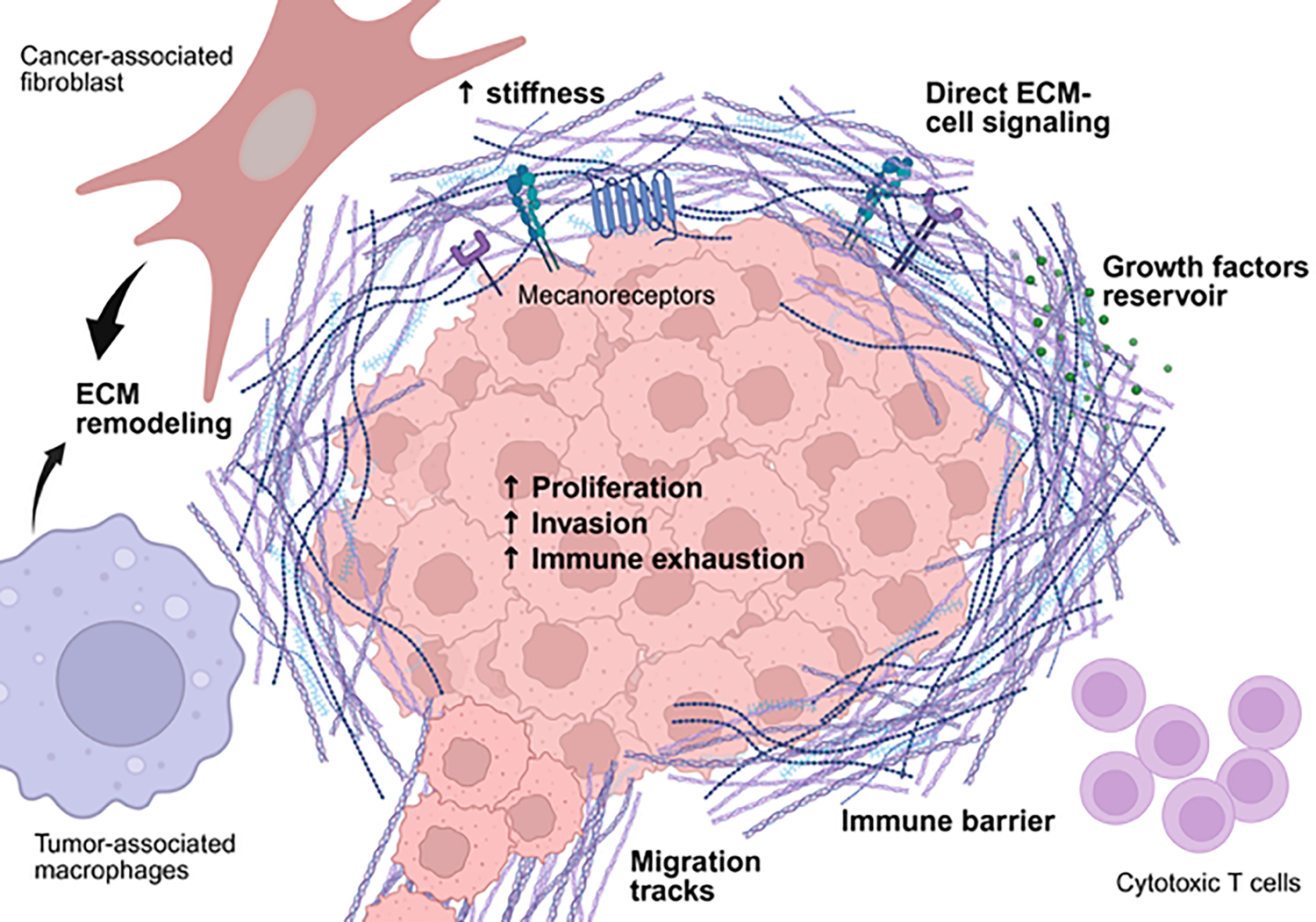
Summary of the different roles of ECM remodeling in cancer progression.

In the TME, the concerted function of fibroblasts and immune cells in response to cancer cell cues (15) actively support proliferation, migration, and invasion of malignant cells, promoting immune exclusion and/or exhaustion and leading to therapeutic resistance (15–19) (Figure 1). Deposition of ECM by CAFs enhances malignant cell mechanotransduction and promotes proliferation and invasion (20, 21). Tumor-associated macrophages (TAMs) also secrete matrix-degrading enzymes, such as metalloproteinases (MMPs) that contribute to ECM remodeling and further promote tumor progression (22, 23), and TGF-β family members, which further enhance CAF activation and immunosuppressive functions (24). Mechanosensing molecules at the tumor membrane convert mechanical cues induced by the ECM into intracellular signals. Key sensors include integrins, mechanosensitive ion channels, and G protein–coupled receptors (25). Direct activation of integrins and discoidin domain receptors by ECM molecules also induces signals controlling tumor progression, therapeutic resistance, and cancer cell heterogeneity (26).

The ECM is composed of complex modular proteins that not only function as structural scaffolds, but also bind and store growth factors that drive short-range paracrine signaling. For example, heparan sulfate proteoglycans bind Wnt family members and other soluble mediators, like cytokines, enhancing Wnt/β-catenin signaling (27). Thus, the ECM also serves as a trap for growth factors, leading to growth factor accumulation that actively supports tumorigenesis (28). Another layer of complexity in the regulatory functions of the ECM relies on matricryptins (29, 30), which are released upon partial proteolysis of ECM components and that, in turn, regulate tumor cell proliferation and migration (31). Matricryptins may function as decoy molecules interfering with ligand-receptor interactions at the cell surface. For example, endostatin and FZC18, two polypeptides excised from the C-terminus and N-terminus, respectively, of collagen type XVIII, inhibit tumor growth in preclinical models by complementary mechanisms. Endostatin binds integrin receptors on endothelial cells, thus inhibiting angiogenesis (32). FZC18 localizes within the pericellular ECM, where it binds Wnt ligands and heterodimerizes with cell surface FZD1 and FZD8 receptors, blocking Wnt-FZD receptor interactions, thereby inhibiting the Wnt/β-catenin pathway (31), and reducing tumor cell proliferation and growth (33).

Modifications of the mechanical properties of the ECM, such as elasticity and stiffness, also promote cancer cell invasion, especially since cancer cells can use fibrillar ECM as migration tracks (34–36). Although ECM remodeling may restrict tumor cell dissemination by creating physical barriers preventing their escape (37), the same barrier can also prevent T cell priming and infiltration through the tumor stroma thus promoting immune exclusion (38, 39). Indeed, ECM stiffness and resistance to the infiltration by immune cells relies on two major family of enzymes, lysyl oxidases (LOXs) and transglutaminases (TGs). The LOX family is composed of five secreted copper-dependent enzymes: LOX and four LOX-like proteins (LOXL1–LOXL4) that catalyze the oxidative deamination of lysine and hydroxylysine in collagen and elastin, leading to covalent cross-linking, resulting in tight lateral stacking of fibers. In turn, TGs catalyze the Ca^++^-dependent formation of covalent bonds between glutamine and lysine, changing the biomechanical properties of the ECM, including type III collagen, fibronectin, and elastin, thus increasing ECM stiffness and resistance to protease degradation (40). In normal development and wound healing, crosslinking enzymes confer tensile strength and structural integrity to tissues. For example, the rate at which a surgical incision restores its tensile strength serves as a benchmark for monitoring postoperative recovery. However, in chronic fibroinflammatory diseases and cancer, this process evolves as recurrent bouts of tissue injury and repair, thus leading to desmoplasia (41, 42).

The ECM mainly exists in two forms: interstitial matrix and basement membranes (43). In both cases, these structures are composed of supramolecular assemblies of a variety of ECM proteins, including collagens, noncollagenous glycoproteins, hyaluronans, and proteoglycans (44). Among the noncollagenous glycoproteins, matricellular proteins, a subset of noncollagenous glycoproteins (such as TNC and POSTN), are ECM-associated proteins that modulate cell-matrix interactions, rather than contributing to structural support. Their interactions with growth factors, integrins, and other ECM components regulate cell adhesion, migration, proliferation, and signaling, with crucial contributions to the TME (45).

The interstitial matrix is mainly composed of fibrillar collagens, fibronectin 1, and elastin, and basement membranes are sheet-like structures rich in nonfibrillar collagens, such as collagen type IV, which form lattices with laminins and nidogens that separate endothelial, epithelial, muscular, or neural cells from the underlying interstitial ECM (43). Beyond these simplified molecular compositions, these structures follow tissue and organ specificities and changes during development and carcinogenesis (46). For example, hyaluronan and proteoglycan link protein (HAPLN1) is an essential component of cartilage, and deletion of this protein in mice leads to impaired development of long bones (47); however, de novo expression of HAPLN1 observed in CAFs in pancreatic cancer and HCC promotes epithelial-mesenchymal transition (EMT), stemness, and immunomodulation (48, 49).

## The matrisome and CAFs

The emerging knowledge on the remodeling of the ECM structure and composition described above underscores the dynamic nature of the cancer ECM, including its assembly and processing by ECM-associated enzymes that are hallmarks of tumor progression in most cancers (50). Expression and functions of these enzymes are the result of crosstalk between cancer cells, endothelial cells, CAFs, TAMs, and other myeloid-derived tumor-infiltrating cells, such as neutrophils. This immune milieu is defined as the fibroinflammatory microenvironment of cancer (15).

The concept of “matrisome analysis” emerged about 15 years ago from the work of Richard Hynes and Alexandra Naba (51–54). To further investigate the mechanisms of ECM remodeling in cancer, their concept was to apply mass spectrometry on ECM-enriched samples to fully dissect the composition of ECM in healthy and diseased tissue at the protein level. To this end, methods had to be developed to solubilize supramolecular ECM protein assemblies, which are otherwise rather insoluble (55). This work gave rise to the concept of a “matrisome” composed of over 1,000 different proteins that are categorized into core matrisome and matrisome-associated proteins. The core matrisome comprises approximately 300 genes encoding proteins that contribute to the structure of the ECM scaffold, primarily fibrillar collagens, glycoproteins, and proteoglycans (53). Matrisome-associated proteins include ECM-affiliated proteins (e.g., collagen-related proteins, transmembrane proteoglycans, matricellular proteins), ECM regulators and modifiers (e.g., LOXs, TGs, and MMPs), and secreted factors that bind to the ECM (e.g., TGF-β, Wnts, and cytokines).

Matrisome analyses of different cancers were compiled in The Matrisome Project, which gathered datasets from 42 studies on the ECM of 39 normal tissue types and eight cancer types (56). Hundreds of proteins were identified, raising the conclusion that the matrisome of cancer largely extends beyond collagens (57). Furthermore, additional studies performed quantitative proteomics to compare tumors with nontumor tissue samples from the same resection specimen. These studies established a footprint of the cancer-specific matrisome (58, 59). Integrative analyses of matrisome in cancer, including genomics, epigenomics, transcriptomics, and proteomics, revealed a widespread upregulation of matrisome genes, with some genes showing patterns of expression more specific to certain subsets of cancer (60). Both fibrillar and basement membrane collagens appear to be increased in most cancers. Some ECM proteins, like the ECM regulator LOX, have a mixed expression pattern. LOX is increased in breast and pancreatic cancer but decreased in some other cancers, like bladder cancer. Among the glycoproteins, osteopontin (SPP1) is increased in a wide variety of solid tumors, including breast, lung, colorectal, liver, and pancreatic cancers. Its expression also correlates with advanced disease and poor prognosis in multiple cancers (61). Functionally, SPP1 promotes tumor growth by enhancing EMT and cell cycle progression (62, 63). Moreover, SPP1 was also shown to play a central role in immune exhaustion by driving an immunosuppressive phenotype in TAMs (64). Yuzhalin and colleagues established a SPP1-containing matrisome-based signature of nine genes that was significantly upregulated across cancers and associated with poor outcomes (65). Nevertheless, mass spectrometry–based matrisome analyses generally fail to capture SPP1 because of its high solubility and small size, leading to its loss along ECM enrichment procedures (55).

## Cancer-specific fibrosis in the matrisome of HCC

Matrisome analysis is particularly relevant in organs where a fibroinflammatory background is an important risk factor for the emergence of cancer, like in HCC, the main type of liver cancer. As mentioned earlier, about 80% of HCCs arise in a backdrop of chronic fibroinflammatory liver disease (66). Several studies analyzed cancer-associated liver fibrosis in mouse and human tissues by qualitative proteomics and identified a wide variety of proteins within the matrisome (67, 68). However, the pathological analysis of HCCs showed that this cancer type is mostly composed of epithelial cells and that CAFs seem to be less present than in other cancer types (69). HCC frequently presents as a soft cellular tumor, especially in comparison with the surrounding nontumor fibrotic tissue. At the same time, HCCs can harbor focal fibrous areas associated with the expression of stem cell markers, cell proliferation signatures, and poor outcomes (70). This histological feature of HCC was termed “fibrous nest” and needs to be distinguished from the scirrhous subtype, which represents 4%–7% of HCCs (71, 72). In the scirrhous subtype, a dense fibroinflammatory stroma affects over 50% of the tumor; by contrast, fibrous nests represent <40% of the tumoral field (73).

A quantitative matrisome analysis of HCC fibrous nests using tandem mass tag mass spectrometry in comparison with the surrounding liver cirrhosis (59) identified more than 200 different HCC-associated ECM proteins, including 31 different types of collagens. By comparing the relative abundance of these proteins between HCC harboring a fibrous nest and cirrhosis, 144 ECM proteins with different levels in between tissue types were identified (59). Moreover, integration of proteomics data with transcriptomics datasets showed that this cancer-specific ECM remodeling strongly overlapped with the previously described Wnt/TGF-β molecular subclass (59). Wnt/TGF-β HCCs (also known as Hoshida’s S1) (74) have been shown to be associated with inflammation, immune exhaustion, and poor survival (75). The link between intratumoral fibrosis and TGF-β signaling activation is well-established, given TGF-β’s central role in CAF activation (76); likewise, the association of a fibrotic phenotype with immune escape is consistent with the mechanisms outlined above (77). Furthermore, fibrous nests likely promote immunotherapy resistance, as suggested in a study from Liu and colleagues (38). Authors analyzed spatial patterns associated with immune checkpoint inhibitor (ICI) resistance in HCCs using single-cell RNA sequencing (scRNA-seq) combined with spatial transcriptomics. They identified a pattern of fibrosis surrounding tumoral cells (a phenotype called “immune barrier”) that was rich in CAFs and in TAMs expressing SPP1 and present only in patients resistant to therapy.

An association between tumor fibrosis and ICI resistance in HCC was also suggested in a recent article that combined spatial transcriptomics and proteomics on biopsies collected before and after immunotherapy and used deep learning to establish a spatial “fingerprint” of treatment resistance (78). The authors concluded that ECM disassembly around the tumor associates with treatment response and that immune-excluding fibrotic ECM and TGF-β signaling associate with resistance. Therefore, cancer-specific ECM remodeling appears to be present in HCCs harboring fibrous nests, which is associated with resistance to immunotherapy.

CAF subclasses as potential drivers and source of cancer-specific fibrosis.

Although CAFs have been traditionally viewed as a homogeneous population within the TME, recent methodological advances have revolutionized our understanding of CAF biology. Advanced methodology applied to multiple solid tumors has uncovered remarkable cellular heterogeneity within CAF subpopulations. These CAF subpopulations were associated with different transcriptomic programs and specialized roles in ECM remodeling, immune modulation, and tumor support (79). Beyond descriptive profiling, several studies were able to associate specific CAF subclasses with clinical features, including patient outcome and therapeutic response (38, 80–82). More recently, large-scale integrative efforts have analyzed CAFs across cohorts of different cancers to propose consensus frameworks or CAF atlases, aiming to unify the classification of fibroblast heterogeneity across tumor types (83–85).

Consensus CAF subclasses traditionally include myofibroblastic CAFs (myCAFs), characterized by high levels of smooth muscle actin (ACTA2), POSTN, and fibroblast activation protein α (FAP); inflammatory CAFs (iCAFs), expressing IL-6, CXCL12/CXCL14, and complement factors; antigen-presenting CAFs, expressing MHC-II molecules such as HLA-DR and CD74; and vascular CAFs, expressing MCAM. These subclasses were consistently identified in scRNA-seq datasets from small cohorts of a single cancer type across studies, but the different CAF atlases performed in larger datasets were able to add more granularity to this classification. For example, Gao and colleagues, via analysis of 517 samples from 11 tissue types, identified up to 20 fibroblast subclasses, including three subtypes of myCAFs and three subtypes of iCAFs (84). Similarly, Liu and colleagues combined the analysis of 1,116 samples from 73 studies, involving 10 cancer types, and ended up with 18 CAFs subclasses (85). Several of these studies support the existence of a subclass of Pi16^+^ progenitor fibroblasts. Interestingly, several markers of the cancer-specific fibrosis of HCC, such as ANXA1 and FBN1, are among the markers of Pi16^+^ CAFs identified by Gao et al. (84). Matrisome analysis combined with scRNA-seq in colorectal cancer also identified a cancer-specific matrisome signature expressed by a subpopulation of Pi16^+^ CAFs associated with poor outcome (58). Another early fibroblast atlas across tissue identified a subtype of Pi16^+^ cells with progenitor properties (86). Finally, a third CAF atlas that included data from 532 samples in 15 tissue types identified Pi16^+^ steady-state-like CAFs (83). Overall, the role and the exact phenotype of these Pi16^+^ CAFs need further investigation regarding their potential importance in supporting tumor-associated fibrosis. Overall, the integration of matrisome data with CAF single-cell atlases provides an opportunity to advance our understanding of the mechanisms underlying immune escape and therapeutic resistance.

## Toward therapeutic targeting of cancer-specific ECM and CAFs

Considering the central role of desmoplasia in cancer and the evidence mentioned above, CAFs have naturally emerged as attractive therapeutic targets (Table 1). Several strategies have been developed to disrupt CAF function, including direct depletion, phenotypic reprogramming, and inhibition of CAF-derived signaling pathways (87). For example, selective depletion of LRRC15^+^ myCAFs was able to enhance the function of CD8^+^ T cells, rendering them more effective in response to anti–PD-L1 treatment in a mouse model of pancreatic cancer (88). Among the CAF-derived targets with the most clinical potential, FAP has shown strong preclinical efficacy. FAP upregulation is a hallmark of CAFs (89), with potential as a unfavorable prognosis biomarker for various cancers (90–92). A number of treatments targeting FAP^+^ CAFs have progressed into clinical trials (Table 1) (87). FAP-targeted radiopharmaceutical 177Lu-EB-FAPI showed encouraging therapeutic efficacy (25% of partial response) and acceptable side effects in a pilot dose-escalation study in metastatic thyroid cancer (93). PDGFR, another key CAF receptor, is targeted by tyrosine kinase inhibitors, such as imatinib and regorafenib (94–96). High expression of PDGFR in tumor stroma is associated with risk of recurrence in several cancers (97–99). While a combination of imatinib and ipilimumab is well-tolerated in patients with advanced cancer, the effects are not synergistic (94). Phase II studies involving the combination of imatinib with other ICIs, such as atezolizumab and pembrolizumab, are currently underway (95). Other avenues may include targeting mechanotransduction (100, 101). As strategies targeting CAFs may blur communications between cancer cells and the TME, unleashing antitumor immunity and leading to transient slowdowns in cancer progression, they may synergize with other approaches. Another strategy to target CAFs is to focus on the secreted factors that comprise the matrisome of cancer, such as cancer-associated fibrosis markers, each of which show potential as clinical biomarkers or as therapeutic targets. Here, we review a selection of five ECM proteins with a robust overexpression in cancer, associated with patient survival and potential as therapeutic targets.

ANXA1, a calcium-dependent phospholipid binding protein that binds to different molecules such as formyl peptide receptors and heparan sulfate (102), is upregulated in multiple cancers, including triple-negative breast cancer, cholangiocarcinoma, esophageal squamous cell carcinoma, bladder cancer, glioma, gastric cancer, papillary thyroid carcinoma, and melanoma, where its expression often correlates with advanced stage and poor prognosis (103–108). Functionally, it promotes cancer cell proliferation, invasion, and metastasis through regulation of EMT and activation of the IL-6/JAK2/STAT3 pathway (106, 109). In the TME, ANXA1 enhances immune exhaustion by driving TAM polarization toward an immunosuppressive phenotype, supporting Treg activity, and upregulating PD-L1 expression at the surface of tumor cells (110, 111). It also facilitates angiogenesis via FPR-mediated VEGF signaling and endothelial activation, while its interaction with CAFs contributes to ECM remodeling and stemness in tumors (112).

FBN1, a major structural glycoprotein of the ECM, is a large, cysteine-rich protein with multiple EGF-like motifs arranged in tandem and TGF-β binding modules (113). FBN1 is upregulated in a wide variety of cancers, including colorectal, prostate, and renal clear cell carcinoma, where its high expression frequently correlates with advanced stage, metastasis, and poor survival (114–117). Mechanistically, FBN1 contributes to tumor progression by direct activation of five different integrins (including α5β1 and αvβ3) and TGF-β receptors. In gastric cancer, FBN1 drives tumor growth by activating PI3K/AKT and focal adhesion kinase pathways (118). In ovarian cancer, it regulates glycolysis and angiogenesis through the VEGFR2/STAT2 and promotes metastasis (116).

Fibronectin 1 is a large, multidomain glycoprotein present in soluble form in the blood and as fibrillar networks in tissues, composing the majority of noncollagenous interstitial matrix (50). Fibronectin 1 overexpression strongly contributes to stiffening and to the formation of migration tracks during cancer invasion (119). Fibronectin 1 is increased in breast, colon, head and neck, liver, and ovarian cancer (60, 120). Tumor cell binding to fibronectin 1 is mediated by integrins (11 different integrins) (121) and syndecans, triggering bidirectional mechanical signaling between the ECM and the cytoskeleton and inducing PI3K and YAP/TAZ signaling (120). Fibronectin 1 also regulates angiogenesis by binding growth factors such as VEGF, supporting tumor neovascularization (122).

POSTN, a secreted matricellular glycoprotein, is one of the most consistently upregulated ECM proteins across human cancers, including breast, lung, pancreatic, liver, prostate, bladder, ovarian cancer, and osteosarcoma (123). Furthermore, high POSTN expression in the tumor stroma or in CAF correlates with advanced stage, metastasis, and poor survival in several cancer types (124–126). Mechanistically, POSTN binds integrins (αvβ3, αvβ5, α6β4) to activate FAK/PI3K/AKT and NF-κB signaling, thereby promoting cancer cell proliferation, survival, EMT, invasion, and metastasis (127). POSTN also contributes to ECM stiffness, generating niches that facilitate tumor cell development and migration (128, 129). Importantly, POSTN also supports angiogenesis by enhancing VEGF signaling (130) and fosters immune exhaustion by recruiting TAMs and Tregs (131–133).

TNC, a large hexameric glycoprotein, is markedly upregulated in the tumor stroma in most epithelial cancers, such as breast cancer, ovarian cancer, pancreatic cancer, colon cancer, or gastric cancer (134). High TNC expression typically localizes at the invasive front of tumors and correlates with advanced stage, metastasis, and poor prognosis (135–137). TNC consists of four different domains that interact with more than 25 different molecules, including EGF receptors, PDGF, FGF, TGF-β, and TLR4 (138). Functionally, TNC promotes tumor progression through multiple mechanisms: it enhances ECM stiffness (139), induces tumor migration and invasion via interaction with integrins (αvβ1, αvβ6 and α9β1) (140), and supports EMT (141) and metastatic dissemination (142). Within the TME, TNC-rich zones are associated with immune exclusion (143), as TNC inhibits T cell proliferation through interaction with α5β1 and αvβ6 integrins (144) and promotes immunosuppressive macrophages via TNF signaling (145).

All the above-mentioned markers have potential for clinical application as biomarkers or therapeutic targets. Several studies suggest the potential of ANXA1 as a diagnostic biomarker for cancers like breast cancer or HCC (146, 147). Methylation of FBN1 in circulating cell-free DNA has also been suggested to be used as a biomarker in colorectal cancer (148). Currently, many efforts are ongoing to develop small molecules targeting fibronectin 1 (149, 150), block fibronectin 1 extracellular domains (151–153), or impair fibronectin 1 binding (154), yet the efficacy of these approaches remains to be demonstrated. This is also the case for TNC, for which preclinical studies have also been performed using Ab-drug conjugates, mAbs, or small peptides (155–157). For ANXA1, FBN1 and POSTN, preclinical studies using specific inhibitors are presently lacking.

## Conclusion

Cancer-associated ECM remodeling has emerged as a central determinant of tumor progression, immune exclusion, and therapeutic resistance. Integrative matrisome analyses, combined with transcriptomic and spatial profiling, have revealed ECM proteins that are consistently enriched across cancer types and associated with poor prognosis in patients. scRNA-seq and large-scale fibroblast atlases suggest that these proteins originate from discrete CAF subpopulations with specialized functions. Together, these findings highlight the dual importance of defining cancer-associated ECM signatures and mapping their cellular sources. A deeper integration of multi-omics datasets with clinical outcomes will be essential to transform matrisome components and CAF subclasses into actionable biomarkers and therapeutic targets, ultimately paving the way for stroma-directed interventions in oncology.

## Author contributions

RD wrote the manuscript and assembled the figure. OM and TFB edited the manuscript.

## Conflict of interest

The authors have declared that no conflict of interest exists.

## Funding support

- European Research Council grants ERC-AdG-2020-FIBCAN 101021417, ERC-2022-PoC1 CANDY 101069276, ERC-2023-PoC FLAMINCO 101122510, ERC-2024-POC 101208554 PACMAN, and EU HORIZON-HLTH-2021-DISEASE-04-07 D-SOLVE 101057917.
- Fondation ARC PANCLAU.
- French National Research Agency RHU DELIVER (ANR-21-RHUS-0001) and LABEX ANR-10-LABX-0028_HEPSYS.
- University of Strasbourg Foundation.
- Alsace Contre le Cancer Foundation.
- Force Foundation.
- Ligue Contre le Cancer, Ille-et-Vilaine, Côtes d’Armor, Vendée (2019; 2024; 2025).
- INSERM, University of Rennes.
- Institut National du Cancer (grant 12688).
- Cancéropôle Grand Ouest (grant STRO-Target).
- Agence Nationale de the Recherche (ANR) grants IdEx Unistra (ANR-10-IDEX-0002), SFRI-STRAT’US project (ANR 20-SFRI-0012), and EUR IMCBio (ANR-17-EURE-0023) under the framework of the French Investments for the Future Program and the France 2030 program.

## Figures and Tables

**Table 1:**
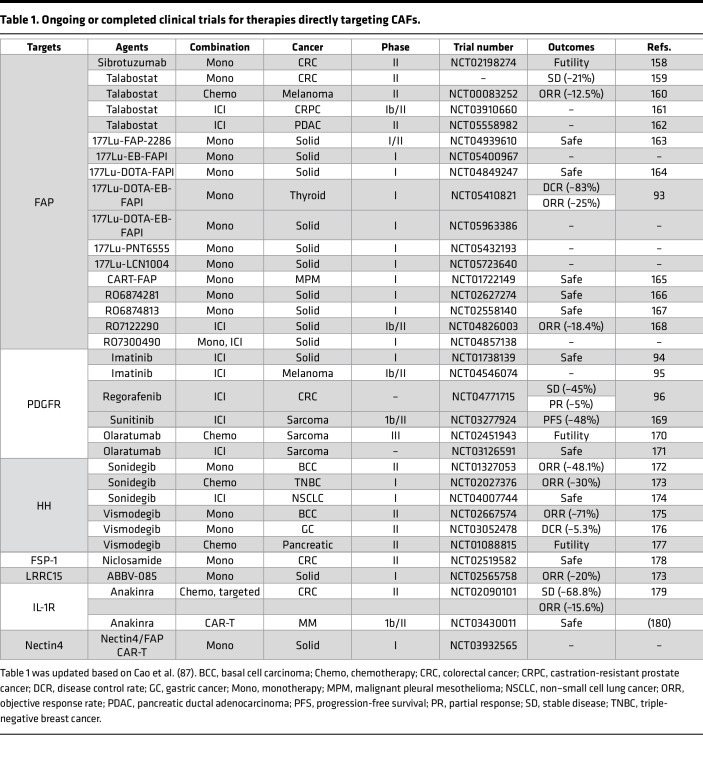
Ongoing or completed clinical trials for therapies directly targeting CAFs.

## Version 1 07/08/2026

Electronic publication
